# Efficacy and safety of a single dose pentamidine (7mg/kg) for patients with cutaneous leishmaniasis caused by *L. guyanensis*: a pilot study*

**DOI:** 10.1590/abd1806-4841.20153956

**Published:** 2015

**Authors:** Ellen Priscilla Nunes Gadelha, Sinésio Talhari, Jorge Augusto de Oliveira Guerra, Leandro Ourives Neves, Carolina Talhari, Bernardo Gontijo, Roberto Moreira da Silva Junior, Anette Chrusciak Talhari

**Affiliations:** 1 Fundação de Medicina Tropical do Amazonas Dr. Heitor Vieira Dourado (FMT-HVD) - Manaus (AM), Brazil

**Keywords:** Leishmaniasis, Cutaneous Leishmaniasis, Pentamidine

## Abstract

**BACKGROUND:**

There have been few studies on pentamidine in the Americas; and there is no consensus regarding the dose that should be applied.

**OBJECTIVES:**

To evaluate the use of pentamidine in a single dose to treat cutaneous leishmaniasis.

**METHODS:**

Clinical trial of phase II pilot study with 20 patients. Pentamidine was used at a dose of 7 mg/kg, in a single dose. Safety and adverse effects were also assessed. Patients were reviewed one, two, and six months after the end of treatments.

**RESULTS:**

there was no difference between the treatment groups in relation to gender, age, number or location of the lesions. Pentamidine, applied in a single dose, obtained an effectiveness of 55%. Mild adverse events were reported by 17 (85%) patients, mainly transient pain at the site of applications (85%), while nausea (5%), malaise (5%) and dizziness (5%) were reported in one patient. No patient had sterile abscess after taking medication at a single dose of 7mg/kg.

**CONCLUSIONS:**

Clinical studies with larger samples of patients would enable a better clinical response of pent amidine at a single dose of 7mg, allowing the application of more powerful statistical tests, thus providing more evidences of the decrease in the effectiveness of that medication. Hence, it is important to have larger studies with new diagrams and/or new medications.

## INTRODUCTION

Leishmaniasis ranks among the world's six most important parasitic disorders. It is considered a neglected disease by the World Health Organization (WHO). It is endemic in 88 countries, and according to estimates, 2 million new cases are diagnosed every year.^1^ Cutaneous leishmaniasis (CL) is the most common form (1 to 1.5 million cases per year) and 90% of patients live in rural areas, in the outskirts of urban areas or in urban areas of seven countries: Afghanistan, Argelia, Brazil, Iran, Peru, Saudi Arabia and Syria.^2^ According to the Brazilian Ministry of Health (BMH), 30.000 new cases are diagnosed every year in Brazil and the prevalent species are *L. braziliensis* and *L. guyanensis*.^3^ In the region of Manaus (western Amazon), *L. guyanensis *is responsible for 95% of CL cases.^4,5,6,7^

In Brazil, meglumine antimoniate (15-20mg/kg/dose intravenously or intramuscularly during 20 days), amphotericin B (1mg/kg daily or on alternating days) and pentamidin isothionate (3 intramuscular doses of 4mg/kg every 72 hours) are the recommended therapies to treat CL.^3^ The efficacy of meglumine antimoniate seems to vary between 26.3%^6^ and 81.6%, regardless of age, disease duration, the number or location of lesions.^7^ In contrast, the effectiveness of amphothericin B ranges from 50 to 100%^8,9^, while pentamidine isothionate has an efficiency rate of 31.2 to 96%.^10,11^

Meglumine antimonial therapy has several side effects including arthralgia, myalgia, anorexia, nausea, vomiting, feeling full, epigastric pain, heartburn, abdominal pain, rash, fever, weakness, headache, dizziness, insomnia, pyrogenic shock and edema.^(3 Am)^ photericin B administration is contraindicated in patients with heart disease, liver disease, and especially nephropathy. The most frequent side effects of anphotericin B are fever, nausea, vomiting, hypokalemia, and phlebitis at the infusion site. Finally, the principal adverse reactions of pentamidine are pain, induration and sterile abscesses at the injection site, as well as nausea, vomiting, dizziness, malaise, myalgia, headache, hypotension, syncope, hypoglycemia and hyperglycemia.^3,5,12^

Pentamidin therapeutic regimen varies considerably in different countries.^13-18^

Roussel *et al*.^18^ (2006) compared 1 and 2 IM injections of pentamidine (7mg/kg) in patients from French Guiana with CL (there is no mention of *Leishmania* species) and obtained cure rates of 78.8%. The increase in the dose from 4mg/kg to 7mg/kg was attributed to the change in pentamidine salt from mesilate (Lomidine^®^ to isothionate. Although Lomidine has long been discontinued, the guidelines from the Brazilian Ministry of Health continue to wrongly recommend the dose of 4mg/kg for isothionate. No study like Russel's has been undertaken in Brazil yet.^19^

Although meglumine antimoniate is regarded as the first-line therapy to treat CL by BMH, in the region of Manaus, pentamidine isothionate has been employed as the first-line drug since 1985.^12^ Neves *et al*.^13^ observed cure rates of 58.1% and 55.5% in patients with CL caused by *L.guyanensis*, treated with 3 IM injections of pentamidine (4 mg/kg every 2 days), and 20 IM injections of antimonials, respectively. The pentamidine regimen is likely to increase adherence to treatment since the short course and small number of injections it requires are an overwhelming advantage, especially for patients from rural and remote areas.

Oral miltefosine has been used in different countries to treat visceral and mucocutaneous leishmaniasis with a cure rate varying between 55 and 94%.^20,21^ Recent reports from Brazil indicate that it is the most effective drug against CL caused by L. guyanensis and L. braziliensis.^4,5^ However, this medication is not commercially available in Brazil.

## MATERIALS AND METHODS

### Ethics Statement

This study was designed in accordance with international ethical guidelines, consistent with the principles originating from the Helsinki Declaration on biomedical research involving human subjects. It was approved by the ethics review committee of the Heitor Vieira Dourado Amazon Tropical Medicine Foundation (FMT-HVD) in Manaus, AM, Brazil. Written informed consent was obtained from all participants prior to their enrollment. In the case of minors, their parents or legal guardians provided written informed consent.

### Study population

This study was conducted from November 2010 through February 2011 at the dermatology outpatient clinic of Heitor Vieira Dourado Amazon Tropical Medicine Foundation (FMT-HVD). Twenty patients of both sexes, aged 16-64, with no more than six CL lesions and three months of evolution, were enrolled in the study.

### Clinical and laboratory workup

All patients underwent pre-assessment via a standardized clinical record form and physical examination. The data collected included details of recent symptoms and past medical history. A full body skin examination was performed by a dermatologist with expertise in leishmaniasis to detect cutaneous lesions. Blood pressure, heart rate, body temperature and weight were recorded before treatment, immediately after drug administration and during follow-up visits. Cutaneous lesions were measured and pictured before treatment. Furthermore, follow-up measurements and pictures were taken one week, one month, two months, and six months after treatment.

The diagnosis of CL was confirmed by a positive skin smear (Giemsa) and skin biopsy of specimens obtained from the border of the lesion. Sections were stained with hematoxylin-eosin and Wade staining for leishmania amastigotes. Species identification was performed through polymerase chain reaction (PCR), as described elsewhere.^14,22^ Two months after treatment, additonal smears were obtained from lesions that were not completely healed and/or showed an increase of at least 50% of its original dimensions.

Other laboratory exams included: complete blood count, capillary blood and venous blood glucose, AST, ALT, creatinine and amylase blood levels, blood urea nitrogen (BUN), stool parasite examination, routine urine examination and rapid test for HIV.

### Drug administration

Pentamidine isothionate (300mg salt per ampoule) was diluted in 5ml of saline solution, and a single intramuscular injection (7 mg/kg) was administered at the outpatient unit of the FMT-HVD.

Patients were given a carbohydrate-enriched meal before treatment to prevent hypoglycemia. They were rested and kept under close clinical observation until one hour after drug administration.

Therapeutic failure was defined as the persistence of clinical signs (onset of new lesions, or more than 50% increase in the size of preexisting lesions), or laboratory findings (positive smears) two months after treatment or anytime during the follow-up period. Rescue treatment with three intramuscular pentamidine injections (4 mg/kg every 72 hours) was prescribed for these patients.

Adverse effects (AE) were classified as mild (drug-related, well-tolerated, and not requiring prescription for symptomatic relief); moderate (drug-related, symptomatic prescription required), and severe (clinically detectable impairment of renal, hepatic or cardiac functions). All adverse effects, regardless of their causality, were noted in the patient's clinical record form.

## RESULTS

Twenty patients (2 females, and 18 males), aged 17-63 years, were included in the study (Table 1). All cases were referred from endemic areas in the outskirts of Manaus and caused by *L. guyanensis*. Most patients (65%) presented with a single lesion and the upper limbs were the most commonly (60%) affected site. Age, gender and weight did not affect cure rates (Table 2).

**Table 1:** Clinical and epidemiological data

		n	%
**Sex**			
	F	2	10.0
	M	18	90.0
			
**Age**			
	< 18	1	5.0
	18 – 36	11	55.0
	36 – 54	6	60.0
	≥ 54	2	10.0
	Mean ± SD	34.5 ± 13.47	
			
**Number of lesions/patient**			
	1	13	65.0
	2	3	15.0
	3	2	10.0
	4	1	5.0
	6	1	5.0
			
**Site of lesion**			
**Head**			
	No	18	90.0
	Yes	2	10.0
**Upper limbs**			
	No	8	40.0
	Yes	12	60.0
			
**Lower limbs**			
	No	14	70.0
	Yes	6	30.0
			
**Trunk**			
	No	16	80.0
	Yes	4	20.0

**Table 2:** Clinical and epidemiological data

		Cure				Total	Odds (IC95%)
		No	%	Yes	%		
**Age**							
	< 18	0	0.0	1	100.0	1	-
	18 - 36	5	45.5	6	54.5	11	
	36 - 54	3	50.0	3	50.0	6	
	36 - 54	1	50.0	1	50.0	2	
	Mean ± SD	36 ± 12.9		33.3 ± 14.4		p-value = 0.494** 0.999*	
							
**Weight**							
	Mean ± SDP	74.2 ± 18.5		70.9 ± 15.1		p-value=0.671	
							
**Gender**							
	F	0	0.0	2	100.0	2	0.0(0.00-6.466)
	M	9	50.0	9	50.0	18	
p-value > 0.9**							

* Fisher Exact Test

** Student T Test

Mild symptoms such as pain at the site of injection (80%), nausea (5%), malaise (5%) and dizziness (5%) were the most frequent complaints. Overall, pentamidine isothionate was well- tolerated by all enrolled patients and no severe adverse effects were detected.

**Figure 1:**
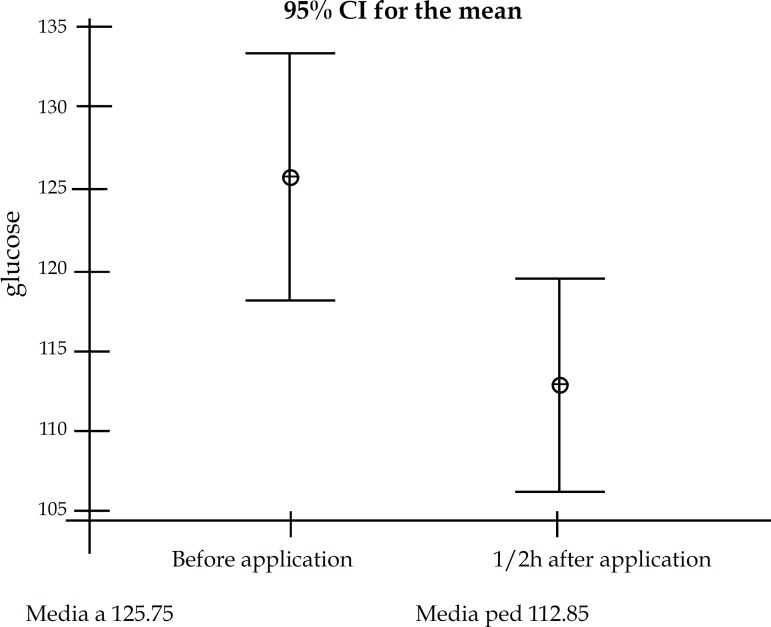
Capillary glucose levels before and 30 minutes after treatment

Increases in AST serum levels were observed in 7 patients (*p*=0.0025) one week after treatment. In all these individuals, normal serum levels were noted one month after treatment (Tables 3 and 4). In 3 patients (*p*=0.002), low capillary blood glucose levels were registered 30 minutes after the injection (Figure 1), with restitution of normal levels during follow-up. No other laboratory abnormalities were detected.

**Table 3:** Laboratory results

		Cure	
**Leucocytes**		**No**	**Yes**
	Mean	6.456	6.636
	SD	2.134	1.574
	N	9	11
	p-value =0.710**		
**Hemoglobin**			
	Mean	15.133	14.7
	SD	0.825	1.485
	N	9	11
	p-value=0.331**		
**Platelets**			
	Mean	252.9	254.7
	SD	59.1	45.6
	N	9	11
	p-value=0.940*		
**Glucose**			
	Mean	97.11	94.36
	SD	6.09	9
	N	9	11
	p-value =0.940*		
**Amilase**			
	Mean	76.3	67
	SD	47.8	18.91
	N	7	10
	p-value =0.999**		
**Creatinine**			
	Mean	1.111	1.1
	SD	0.127	0.2408
	N	9	11
	p-value =0.429*		
**BUN**			
	Mean	26	30.27
	SD	10.05	11.53
	N	9	11
	p-value =0.656**		
**AST**			
	Mean	26.89	23.4
	SD	13.99	7.95
	N	9	11
	p-value =0.720**		
**ALT**			
	Mean	39.56	25.9
	SD	27.64	12.22
	N	9	11
	p-value =0.278**		
**Phosphatase alkaline**			
	Mean	167.2	168.9
	SD	42	35
	N	9	11
	p-value =0.380*		

* Student T Test

** Mann-Whitne Test

**Table 4:** Laboratory results before and after treatment

	Period	N	Mean	SD	p-value
Leucocytes	Before	20	6.555	1.797	0.134*
	After	20	6.91	1.548	
Hemoglobin	Before	20	14.895	1.223	0.15**
	After	20	14.645	1.209	
Platelets	Before	20	253.9	50.7	0.226*
	After	20	260.7	55.7	
Glucose	Before	20	95.6	7.76	0.970*
	After	20	95.5	10.2	
Amilase	Before	15	70.82	32.86	0.172**
	After	15	71.07	20.79	
Creatinine	Before	20	1.105	0.1932	0.223**
	After	20	1.165	0.2412	
BUN	Before	20	28.35	10.83	0.420**
	After	20	29.55	8.69	
AST	Before	19	25.05	11.03	0.025**
	After	19	31.63	18.26	
ALT	Before	19	32.37	21.52	0.074**
	After	19	38.11	27.75	
Phosphatase alkaline	Before	20	168.15	37.23	0.036*
	After	20	163.2	34.11	

* Student T Test

** Wilcox Test

**Figure 2:**
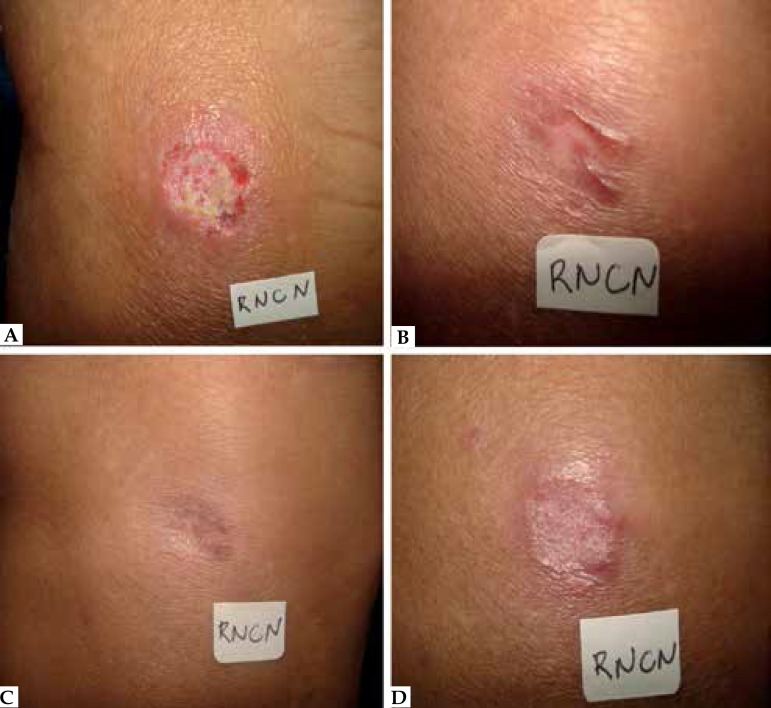
Patients cured. Patient who was cured. Before treatment **(A)**, a month after **(B)**, two months after **(C)** and six months after the end of treatment **(D)**

**Figure 3:**
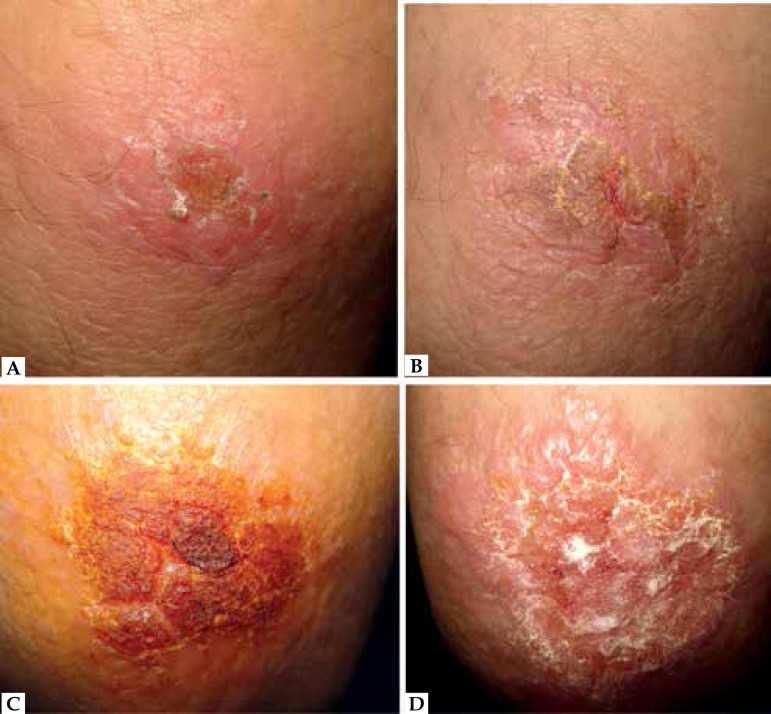
Patient with clinical failure. Before treatment **(A)**, a week after **(B)**, a month **(C)** and two months after the end of treatment **(D)**

After a 6-month follow-up, 11 (55%) patients were considered cured. (Figures 2, 3)

## DISCUSSION

Pentamidine is one of several diamidines with significant anti-parasitic activity. Since it was first synthesized in the early 1940's, it has been widely used in treating human African trypanosomiasis, infection by *Pneumocystis jirovencii* and visceral and cutaneous leishmaniasis. Pentamidine mesilate (Lomidine^®^ has been discontinued and currently only pentamidine isothionate (Pentacarinat^®^, Pentam^®^ is available. Dorlo and Kager^19^ (2008) first pointed out that the recommended dose of mesilate (4mg/kg) ought to be increased to 7 mg/kg when pentamidine isothionate is prescribed. Nevertheless, the BMN guidelines still recommend the 4mg/kg dose.

The range of treatment regimens reported in articles from Latin America precludes any conclusions and renders discrepant cure rates of 35-96%.^14,15,16,17^

Cure rates of 71 and 73.2% were reported in Brazil with three intramuscular injections of pentamidine isothionate (4mg/kg every 2 days) and intravenous meglumine antimonate (20mg Sb^(+5)^/kg/day during 20 days), respectively. The prevalent species of CL in this study was *L. (V.) brasiliensis* (57, 14%).^23^

Roussel *et al*. (2006) obtained a cure rate of 78.8% with a single dose (7mg/kg) in patients from French Guiana, where *L. guyanensis* is prevalent.^18^ Our cure rate (55%) was similar to that found by Neves *et al*. (2011).^12^ Although our rates are lower than those reported by Roussel, it is important to note that they are similar to other regimens, such as the 20 day-treatment with meglumine injections or 3 IM injections of pentamidine (three doses of 4mg/kg every 2 days).^18^ Our data highlight the need for further investigation with more patients.

Common AE from pentamidine include pain at the site of administration, abscess formation, collapse (presumably due to the drug entering a vein), profound weakness, anorexia, nausea, vomiting, abdominal pain, glycosuria, disturbed glucose tolerance tests and frank diabetes, hypotension and headache.^24^

According to Costa, the induction of diabetes mellitus is dose-related and may occur with total doses > 1g.^25^

In 1970, Bryceson reported the presence of diabetes mellitus among 24 patients in Ethiopia treated with pentamidine (daily or on alternate days at a dose of 1.0 mL per 10kg).^24^ The total dose per course was 1-3.4 grams of mesilate pentamidine.

Furthemore, during follow-up for 41 patients with disseminated leishmaniasis, Bryceson (1968) administered courses of 0.8 to 4.g for periods of up to 9 months. Four patients developed diabetes^26^; with total pentamidine dosages of 9.2, 5.6, 3.8, and 2.5g, respectively.

Based on these papers, Bryceson stressed the need for careful use of pentamidine (Lomidine) and suggested that weeekly glucose tolerance tests should be performed whenever the drug is administered.^27^

However, these effects, particularly diabetes, seem to be rare when isothionate is used.^6,7,8^ A plausible explanation is that the total dose rarely exceeds 1g. Transient hypoglycemia right after drug administration was detected in 3 of our patients. Normal glucose levels were observed in the follow-up exams of all patients. Elevation of AST was observed in 7 patients and, again, normal results were obtained during follow-up.

A common adverse effect is the development of an indurated area at the site, frequently followed by abscess formation. No patient in our study presented this side effect, probably because deep IM application is an effective preventive measure.

## CONCLUSIONS

Single dose (7mg/kg) IM of pentamidine isothionate is as effective as the regimen proposed by the guidelines of the Brazilian Ministry of Health (3 IM injections, 4mg/kg every 2 days) for patients with CL caused by L.guyanensis. No severe AE were observed.

It is plausible that single dose pentamidine regimens increase patients' adherence to treatment. This is especially true in a region where patients come from rural and remote areas and experience great difficulty in accessing health facilities. Therefore, pentamidine should be considered an alternative to antimonials when suitable. Nevertheless, further studies with a larger number of patients are required.

## References

[1] World Health Organization (2007). Sixtieth World Health Assembly. Control of leishmaniasis.

[2] Desjeux P (2004). Leishmaniasis: current situation and new
perspectives. Comp Immunol Microbiol Infect Dis.

[3] Brasil, Ministério da Saúde, Secretaria de Vigilância em Saúde, Departamento de Vigilância Epidemiológica (2007). Manual de Vigilância da Leishmaniose Tegumentar Americana.

[4] Chrusciak-Talhari A, Dietze R, Chrusciak Talhari C, da Silva RM, Gadelha Yamashita EP, de Oliveira Penna G (2011). Randomized controlled clinical trial to access efficacy and
safety of miltefosine in the treatment of cutaneous leishmaniasis
Caused by Leishmania (Viannia) guyanensis in Manaus,
Brazil. Am J Trop Med Hyg.

[5] Machado PR, Rosa ME, Costa D, Mignac M, Silva JS, Schriefer A (2011). Reappraisal of the immunopathogenesis of disseminated
leishmaniasis: in situ and systemic immune response. Trans R Soc Trop Med Hyg.

[6] Romero GA, Guerra MV, Paes MG, Macêdo VO (2001). Comparison of cutaneous leishmaniasis due to Leishmania
(Viannia) braziliensis and L. (V.) guyanensis in Brazil: therapeutic
response to meglumine antimoniate. Am J Trop Med Hyg.

[7] Talhari S, Sardinha JC, Schettini APM, Arias JR, Naiff RD (1985). Tratamento da leishmaniose tegumentar americana. Resultados
preliminares com a pentamidina. An Bras Dermatol.

[8] Name RQ, Borges KT, Nogueira LSC, Sampaio JHD, Tauil PL, Sampaio RNR (2005). Clinical, epidemiological and therapeuthic study of 402
patients with american cutaneous leishmaniasis attended at University
Hospital of Brasilia, DF, Brazil. An Bras Dermatol.

[9] Motta JOC (2006). Estudo comparativo da resposta imunológica e clínica entre a
anfotericina B lipossomal e o N-metil Glucamina em pacientes com a
forma localizada da leishmaniose tegumentar americana (LTA).

[10] Thakur CP (2001). A single high dose treatment of kala-azar with Ambisome
(amphotericin B lipid complex): a pilot study. Int J Antimicrob Agents.

[11] de Oliveira Guerra JA, Talhari S, Paes MG, Garrido M, Talhari JM (2003). Clinical and diagnostic aspects of American tegumentary
leishmaniosis in soldiers simultaneously exposed to the infection in
the Amazon Region. Rev Soc Bras Med Trop..

[12] Soto J, Buffet P, Grogl M, Berman J (1994). Successful treatment of Colombian cutaneous leishmaniasis
with four injections of pentamidine. Am J Trop Med Hyg.

[13] Neves LO, Talhari AC, Gadelha EP, Silva RM, Guerra JA, Ferreira LC (2011). A randomized clinical trial comparing meglumine antimoniate,
pentamidine and amphotericin B for the treatment of cutaneous
leishmaniasis by Leishmania guyanensis. An Bras Dermatol.

[14] Pradinaud R (1988). Tegumentary leishmaniasis in French Guiana. Bull Soc Pathol Exot Filiales.

[15] Lai A, Fat EJ, Vrede MA, Soetosenojo RM, Lai A, Fat RF (2002). Pentamidine, the drug of choice for the treatment of
cutaneous leishmaniasis in Surinam. Int J Dermatol.

[16] Andersen EM, Cruz-Saldarriaga M, Llanos-Cuentas A, Luz-Cjuno M, Echevarria J, Miranda-Verastegui C (2005). Comparison of meglumine antimoniate and pentamidine for
peruvian cutaneous leishmaniasis. Am J Trop Med Hyg.

[17] Soto J, Buffet P, Grogl M, Berman J (1994). Successful treatment of Colombian cutaneous leishmaniasis
with four injections of pentamidine. Am J Trop Med Hyg.

[18] Roussel M, Nacher M, Frémont G, Rotureau B, Clyti E, Sainte-Marie D (2006). Comparison between one and two injections of pentamidine
isethionate, at 7 mg/kg in each injection, in the treatment of
cutaneous leishmaniasis in French Guiana. Ann Trop Med Parasitol.

[19] Dorlo TPC, Kager PA (2008). Pentamidine dosage: a base/salt confusion. PLoS Negl Trop Dis.

[20] Soto J, Arana BA, Toledo J, Rizzo N, Vega JC, Diaz A (2004). Miltefosine for New World cutaneous leishmaniasis:
Placebo-controlled multicenter study. Clin Infect Dis.

[21] Soto J, Toledo J, Gutierrez P, Nicholls RS, Padilla J, Engel J (2001). Treatment of American cutaneous leishmaniasis with
Miltefosine, an oral agent Clin Infect. Dis.

[22] Garcia FCB, Santos SSR, Chociay MF, Medeiros ACR, Roselino AMF (2005). Subsidiary methods for the diagnosis of American tegumentar
leishmaniasis (ATL): comparison of sequencing of DNA and PCR-RFLP for
identification of leishmania species in skin samples. An Bras Dermatol.

[23] Paula RCD, Sampaio JHD, Cardoso DR, Sampaio RNR (2003). Estudo comparativo da eficácia de isotionato de pentamidina
administrada em três doses durante uma semana e de N-metil-glucamina
20mgSbV/kg/dia durante 20 dias para o tratamento da forma cutânea da
leishmaniose tegumentar americana. Rev Soc Bras Med Trop.

[24] Bryceson ADM (1970). Diffuse Cutaneous Leishamaniasis in Ethiopia.
II.Treatment. Trans R Soc Trop Med Hyg.

[25] Costa JLM (1993). O uso clínico das pentamidinas com especial referência nas
pentamidinas. Acta Amazônica.

[26] Bryceson A (1968). Pentamidine induced-diabetes mellitus. East Afr Med J.

[27] Bryceson A, Woodstock L (1968). The cumulative effect of pentamidine dimethanesulfonate on
the blood sugar. East Afr Med J.

